# Comparative Evaluation of Portable and Benchtop NIR Spectroscopy and Hyperspectral Imaging for Detecting Honey Adulteration

**DOI:** 10.3390/s26092750

**Published:** 2026-04-29

**Authors:** Aysenur-Betul Bilgin, Miguel Vega-Castellote, José-Antonio Entrenas, Irina Torres-Rodríguez, Didem-Peren Aykas, Pervin Basaran, Dolores Pérez-Marín

**Affiliations:** 1 Department of Food Engineering, Faculty of Chemical and Metallurgical Engineering, Istanbul Technical University, 34469 Istanbul, Türkiye; basaranakocakp@itu.edu.tr; 2 Department of Bromatology and Food Technology, Escuela Técnica Superior de Ingeniería Agronómica y de Montes (ETSIAM), University of Cordoba, Rabanales Campus, 14071 Córdoba, Spain; miguel.vega@uco.es; 3 Department of Animal Production, Ingeniería de Sistemas Agroganaderos (ISAG) Research Group, Escuela Técnica Superior de Ingeniería Agronómica y de Montes (ETSIAM), University of Cordoba, Rabanales Campus, 14071 Córdoba, Spain; p82enlej@uco.es (J.-A.E.); g72toroi@uco.es (I.T.-R.); 4 Department of Food Engineering, Faculty of Engineering, Adnan Menderes University, 09100 Aydin, Türkiye; didem.cinkilic@adu.edu.tr

**Keywords:** honey authentication, NIR spectroscopy, hyperspectral imaging, multivariate classification, chemometrics, PLS-DA, food adulteration

## Abstract

**Highlights:**

**What are the main findings?**

- Portable and benchtop NIR spectroscopy systems, along with hyperspectral imaging, were comparatively evaluated for the detection of honey adulteration.
- Portable and benchtop NIR combined with PCA and PLS-DA achieved 100% classification accuracy, sensitivity, and specificity for distinguishing pure and adulterated honey.

**What are the implications of the main findings?**

- Portable NIR spectroscopy enables the rapid, non-destructive, and on-site authentication of honey, eliminating the need for laboratory infrastructure.
- The method provides a cost-effective screening tool to enhance quality control and combat honey fraud in supply chains.

**Abstract:**

Honey adulteration remains a major challenge for ensuring food authenticity and sustainable quality control. In this study, near-infrared (NIR) spectroscopy and hyperspectral imaging (HSI) were comparatively evaluated as green, non-destructive analytical techniques for the discrimination of pure and adulterated honey using chemometric modeling. A total of 180 honey samples, including pure and adulterated samples with agave syrup, sucrose syrup, or water at varying concentrations, were analyzed using two NIR platforms (MicroNIR™ 1700 and NIRS™ DS2500) and an HSI system (Micro-Hyperspec^®^ NIR camera). Principal component analysis (PCA) and partial least squares discriminant analysis (PLS-DA) were applied for exploratory analysis and supervised classification, respectively. Both techniques enabled effective discrimination between pure and adulterated honey. The results demonstrated that the two NIR platforms achieved superior classification performance: the MicroNIR™ 1700 yielded overall sensitivities, specificities, and accuracies of 100%, respectively. While the HSI system provided complementary spectral-spatial information, its performance and that of the NIRS™ DS2500 were slightly lower, with an overall accuracy of 93.10%, particularly at low levels of adulteration (≤10%). Overall, these results demonstrate that NIR-based spectroscopy is a reliable, fast, non-destructive, and eco-friendly analytical tool for testing the authenticity of honey. The portable NIR system, in particular, provides a cost-effective and field-deployable solution for in situ quality control. Integrating it into routine quality control practices could help prevent food fraud, protect consumer trust, and promote sustainable industry development.

## 1. Introduction

The rapid growth of the global food industry has increased the importance of food authenticity. Food fraud, which encompasses mislabeling, dilution, substitution, and the addition of undeclared substances, not only erodes consumer trust but also disrupts market fairness and compromises public health [1]. Honey, a high-value commodity with a complex composition that makes detection challenging, is frequently adulterated worldwide due to its economic value [2]. A European Union (EU) survey of 320 honey consignments from 20 countries found that 46% were suspected of non-compliance, highlighting the widespread nature of the issue [3]. Despite being the world’s second-largest honey producer after China, with 114,886 tonnes in 2022 [4,5], authenticity remains a major challenge for Türkiye. An EU report [5] found that 93% of Turkish samples were suspicious, compared to 74% of Chinese samples and 100% of UK imports, emphasizing the need for reliable authenticity testing.

Honey, produced by bees from plant nectar, is valued for its nutritional and therapeutic properties, including its antimicrobial, antioxidant, and anti-inflammatory effects [6,7,8]. However, its susceptibility to adulteration with sugar syrups from corn, cane, beet, rice, wheat, or agave [9,10,11,12] is a major concern, given that its main sugars (fructose, glucose, maltose, and sucrose), along with minor amino acids, proteins, vitamins, minerals, and phenolics [13], make it susceptible to adulteration. Such practices, whether through direct blending or indirect bee feeding, reduce the nutritional and economic value of honey [14,15,16].

Although traditional methods of assessing honey authenticity, such as carbon isotope ratio analysis and chromatographic techniques, provide high analytical accuracy, they are often costly and time-consuming and require skilled operators [14]. Furthermore, natural variability among floral sources and the close chemical similarity between authentic honey sugars and common adulterants significantly complicate reliable detection. These limitations highlight the need for rapid, practical, and non-destructive analytical approaches that can ensure honey authenticity while protecting both producers and consumers worldwide.

Spectroscopic techniques, including near-infrared (NIR), Fourier transform infrared spectroscopy, and laser-induced breakdown spectroscopy, as well as hyperspectral imaging (HSI), have emerged as powerful tools for assessing the authenticity of food [17,18,19,20]. Among these, NIR spectroscopy (NIRS), in particular when combined with multivariate analysis, has proven to be a rapid and cost-effective method of honey authentication [21,22,23,24,25]. HSI further enhances detection by integrating spectral and spatial information, enabling the identification of compositional variations and adulteration at the pixel level [20].

Both NIRS and HSI are typically coupled with chemometric and machine learning algorithms, such as partial least squares discriminant analysis (PLS-DA), support vector machines (SVM), k-nearest neighbors (kNN), and neural networks. This is often followed by spectral pre-processing to enhance classification performance. For example, Shao et al. [25] used HSI combined with LIBSVM classification to detect honey adulteration with fructose and sucrose (5–40%), achieving 92.5% accuracy. Similarly, Phillips and Abdulla [26] used HSI with kNN and SVM, achieving accuracies above 95% for both binary and multi-class adulteration detection. Beyond HSI-based approaches, benchtop and portable NIRS systems have also demonstrated robust performance in honey adulteration detection. Bertotto et al. [18] reported that benchtop NIRS, using PLS-DA models with first derivative and standard normal variate (SNV) pre-processing, achieved 100% sensitivity for glucose and rice syrup adulteration, outperforming CNN-ANN models. Whei Miaw et al. [27] achieved 100% classification accuracy using a portable MicroNIR™ 1700 with a one-class soft independent modeling of class analogy (SIMCA) model, incorporating SNV, multiplicative scatter correction, and Savitzky-Golay smoothing preprocessing.

In summary, while previous studies have demonstrated that both benchtop and portable NIRS, as well as NIR-based HSI, can detect honey adulteration with high accuracy, most investigations have focused on a single analytical platform, employed different sample sets, or used varying chemometric approaches. This has limited the possibility of direct comparison. The primary aim of this study is therefore to systematically benchmark multiple NIR configurations, including benchtop and portable instruments, against NIR-based HSI under identical experimental conditions and using a consistent chemometric workflow. This comparison is essential in order to define the operational boundaries between high-precision laboratory analysis and rapid in situ screening. By evaluating classification sensitivity at low adulteration levels (≤10%) and assessing practical applicability, this research provides stakeholders in the honey industry with a clear decision-making framework, identifying the most robust and cost-effective tool for routine quality control. Thus, the study bridges the gap between laboratory-based research and real-world, in-field honey authenticity control.

## 2. Materials and Methods

### 2.1. Sampling

A total of 60 pure honey samples were used in this study, collected from diverse geographical regions between 2023 and 2025 to ensure representative variation in botanical and environmental origins (Table 1).

The samples were obtained from local beekeepers. All samples were stored in plastic bottles at room temperature until analysis.

For honey adulteration, two types of agave syrup, one sucrose syrup, and laboratory-grade distilled water were used as adulterants. The agave syrups were purchased from local markets in Córdoba (Spain). Two commercial products were selected to represent different color and compositional profiles: light agave syrup (Azucarera, Spain; 69 g of sugars, 1207 kJ/284 kcal per 100 g) and dark agave syrup (Eco Wassy, Spain; 73.9 g of sugars, 1292 kJ/304 kcal per 100 g). Both were produced from Agave azul cultivated using organic farming in Mexico. The sucrose syrup was prepared by dissolving 350 g of sucrose in 350 g of distilled water [28], heating the mixture between 100 and130 °C until complete dissolution, and then cooling it to room temperature. All syrups and distilled water were stored in sterile plastic bottles at room temperature prior to use. 

Adulteration levels were established based on the weight percentage (*w*/*w*%) of the adulterant (syrups or water) relative to pure honey. Ten pure honey samples were selected from the initial collection to prepare adulterated mixtures. For agave syrup adulteration, both light and dark agave syrups were blended with honey at concentrations of 5%, 10%, 20%, 30%, 40%, and 50% (*w*/*w*). Similarly, sucrose syrup adulteration was prepared at the same concentration levels. For distilled water adulteration, mixtures were prepared at 5%, 10%, and 20% (*w*/*w*). A summary of the number of pure and adulterated honey samples used in this study is shown in Table 2.

All mixtures were gently stirred at room temperature to ensure homogeneity. Mixing continued until a visually uniform solution was obtained. Prior to spectral collection, the samples were incubated overnight at 40 °C in an incubator to dissolve any crystals present.

### 2.2. Instrumentation and Spectra Acquisition

#### 2.2.1. NIR Spectra Acquisition

Near-infrared spectra were acquired using two instruments: a portable MicroNIR™ Pro 1700 (VIAVI Solutions, Inc., Santa Rosa, CA, USA) and a benchtop FOSS NIRS™ DS2500 (FOSS, Hillerød, Denmark).

**Figure 1:**
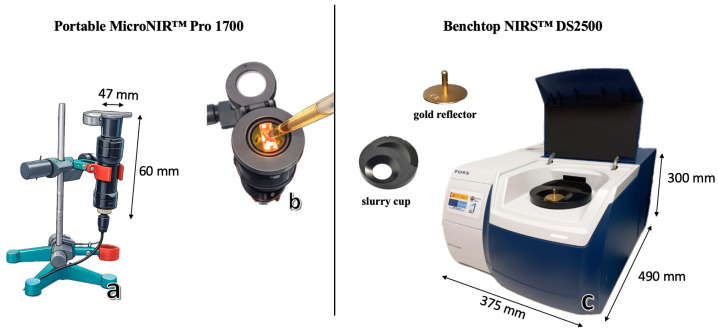
Measurement configurations used for honey adulteration analysis: portable MicroNIR™ Pro 1700 with liquid sampling setup (**a**,**b**) and benchtop NIRS™ DS2500 equipped with a slurry cup and gold reflector (**c**).

The MicroNIR™ Pro 1700 is based on linear variable filter (LVF) technology and operates within the 908–1676 nm wavelength range, with a spectral resolution of 6.2 nm. It is also equipped with a 128-pixel indium gallium arsenide (InGaAs) photodiode array detector. Measurements were performed in transflectance mode using a liquid-sample accessory (Figure 1a,b). Each spectrum was recorded with an integration time of 8 ms and 100 scans per measurement. Instrument performance was validated every 10 min using white and dark reference standards. Spectral acquisition was carried out using MicroNIR™ Pro software version 2.2 (VIAVI Solutions, Inc., Santa Rosa, CA, USA).

The FOSS NIRS™ DS2500 spectrometer has a broader spectral range (400–2500 nm) and a spectral resolution of 2 nm. It is equipped with both a silicon detector (400–1100 nm) and a lead sulfide detector (1100–2500 nm). The instrument operates in reflectance mode. A DS2500 slurry cup and gold reflector were used as the background (Figure 1c) to collect transflectance spectra. The transflectance mode was chosen to accommodate the semi-translucent nature of honey. With the gold reflector in place, the NIR is permitted to pass through the sample twice, effectively doubling the optical path length. This enhances the interaction between the incident light and the internal O-H and C-H overtones, resulting in more representative and sensitive spectral data for adulteration detection. Spectral acquisition was performed using ISIscan NOVA 2022 software (FOSS Analytical A/S, Hillerød, Denmark).

Three and two spectra were collected per sample with the portable and benchtop instruments, respectively. These were averaged to obtain a mean spectrum per sample. The reflectance data were transformed into absorbance as Log(1/Reflectance). To ensure instrumental stability, the system automatically measured the internal white reference.

#### 2.2.2. Hyperspectral Imaging (HSI): Image Acquisition

**Figure 2:**
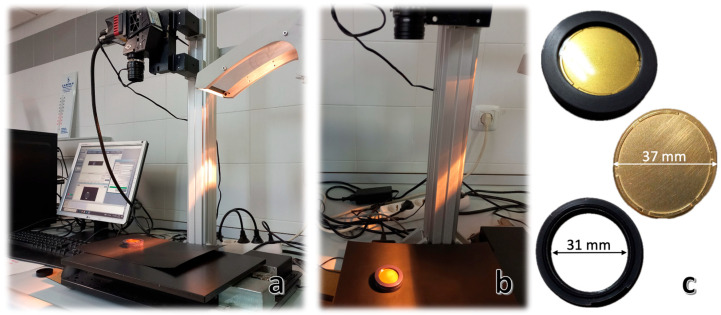
Hyperspectral imaging setup and accessories. (**a**) A laboratory hyperspectral imaging system comprising a camera, an illumination unit, and a computer interface. (**b**) A close-up view of a honey sample positioned under the hyperspectral camera for measurement. (**c**) A gold reflector, a glass cup, and a sample between the gold reflector and the glass cup are used for calibration.

Hyperspectral images (HSI) were acquired in reflectance mode using a laboratory-based push-broom system (Figure 2a,b). This consisted of a Micro-Hyperspec^®^ NIR camera (X-series, Headwall Photonics, Inc., Bolton, MA, USA), which operated over the 889–1702 nm range. It had an 8 mm length lens, 320 spatial pixels, and an InGaAs sensor that captured 67 spectral bands.

An integrated lighting module provided uniform illumination, and a motorized translation stage conveyed samples across the camera’s field of view at a constant speed of 32.65 mm/s. Each hyperspectral image consisted of 200 lines, with 318 pixels per line. Data acquisition was performed using the Hyperspec III Software version 5.5.1 (Headwall Photonics Inc., Fitchburg, MA, USA).

For spectral collection, honey samples were evenly spread between a glass cup and a gold reflector (Figure 2c) to enable transflectance measurements. Prior to acquisition, calibration was conducted using both a dark reference (where the camera lens was covered to prevent any light from reaching the sensor) and a white reference (a 99% reflectance panel; Spectralon™, SRS-99-10, Labsphere, Inc., North Sutton, NH, USA).

As with the NIR data, the reflectance data acquired were converted to absorbance as Log(1/Reflectance) for subsequent analysis.

In summary, the instrumental properties of the three devices are summarized in Table 3.

### 2.3. Data Analysis

Data acquired from the portable and benchtop NIR devices and the HSI system were analyzed using a standardized chemometric workflow. First, spectral data were subtracted from the HSI images using image processing techniques to isolate representative spectral information for each sample. The spectral data datasets obtained from all three instruments were then preprocessed and analyzed using multivariate statistical methods. All data processing and chemometric analyses were conducted in MATLAB (version R2024a; MathWorks, Natick, MA, USA) equipped with the PLS Toolbox version 9.5.1 (Eigenvector Research Inc., Manson, WA, USA).

#### 2.3.1. Spectral Data Extraction from HSI Images

Flat-field corrections were applied to achieve a uniform photometric response per pixel. The reflectance value (R) for each pixel was then calculated using the standard flat-field correction formula (Equation (1)) [28].

(1) $$R=\frac{I_{sample}-I_{dark}}{I_{white}-I_{dark}}$$

where *I_sample_* is the raw intensity of the sample, *I_dark_* is the dark reference, and *I_white_* is the white reference. 

Following correction, regions of interest (ROIs) were manually selected within each image to isolate homogeneous honey areas and exclude background regions and artifacts related to the edges (e.g., shadows or cup reflections) inherent to circular sample holders. The selected ROIs were then unfolded from the 3D hyperspectral cubes (rows × columns × wavelengths) into 2D matrices (pixels × wavelengths). A mean spectrum for each sample was calculated by averaging the spectral data across all pixels in the ROIs. To minimize spectral noise and enhance signal quality, only the 948–1690 nm wavelength range, corresponding to 63 spectral variables, was used for further analysis.

#### 2.3.2. Exploratory Analysis of the Spectral Data

An exploratory analysis of the spectral data acquired from each device (portable and benchtop NIR instruments and HSI camera) was first conducted using a PCA. The spectral data were preprocessed to enhance signal quality and reduce scattering effects by applying standard normal variate (SNV) and detrending (DT) methods [29], followed by a first derivative treatment and mean centering. Potential spectral outlier samples were identified by assessing the Q residuals and Hotelling’s *T*^2^ statistics [30]. Since outliers can negatively impact model accuracy, they were identified and removed if their removal was justified. The confidence limits for both Q residuals and Hotelling’s *T*^2^ were set at 95%.

#### 2.3.3. Development of Discriminant Models for the Classification of Pure and Adulterated Honey

Once spectral outliers were removed, samples were systematically selected based on their spectral distances from the population center, ordered from smallest to largest, to construct the calibration and validation sets, following the method proposed by Shenk and Westerhaus [31]. This structured selection ensured that the calibration and validation sets were representative of the full spectral variability within the dataset for each instrument. To maintain class independence and prevent sample overlap, ten pure honey samples were dedicated exclusively to the preparation of adulterated samples and were excluded from the pure honey class.

To ensure a balanced distribution, consistent group proportions were maintained: one out of every six samples (~20%) was allocated to the validation set (*n* = 29), while the remaining ~80% formed the calibration set (*n* = 140). Specifically, a 2:1:1 ratio was preserved for sucrose, agave, and water-adulterated samples in both subsets (50:25:25 in calibration and 10:5:5 in validation), alongside a proportional number of pure honey samples. This approach was applied to the complete set of honey spectra acquired with MicroNIR™ 1700. The same sample sets were then selected for the Micro-Hyperspec^®^ NIR camera and the NIRS™ DS2500, allowing direct comparison of the results.

Pure and adulterated honey samples were classified using Partial Least Squares-Discriminant Analysis (PLS-DA), a supervised multivariate classification method [32,33,34,35]. Spectral data were pre-processed to minimize undesirable effects such as noise, baseline shifts, scattering, and path length variations, while enhancing chemical information relevant for discrimination. The evaluated spectral pre-processing techniques to optimize model performance included multiplicative scattering correction (MSC), Savitzky-Golay (SG) derivative, standard normal variation (SNV), detrending (DT), and mean center (MC). Some combinations of these techniques were tested to determine the most effective preprocessing strategies for each instrument. SNV and DT were applied for scatter correction [29]. SG smoothing was used to reduce random noise, remove vertical offsets, and correct linearly sloping baselines, and MC was applied to remove common information from the spectra to enhance variation relevant to classification [36,37].

All classification models were built using 8-fold cross-validation with the Venetian-blind approach to ensure balanced and systematic representation of the sample population across the folds. The performance of the cross-validation models was evaluated using the following metrics: sensitivity (true positive (*TP*) rate or recall), specificity (true negative (*TN*) rate), and accuracy [38]. To calculate accuracy in a classification, the following formula (Equation (2)) was used:

(2) $$Accuracy\;(\%)=\frac{TP+TN}{TP+TN+FP+FN}$$

where *TP* is true positives, *TN* is true negatives, *FP* is false positives, and *FN* is false negatives.

**Figure 3:**
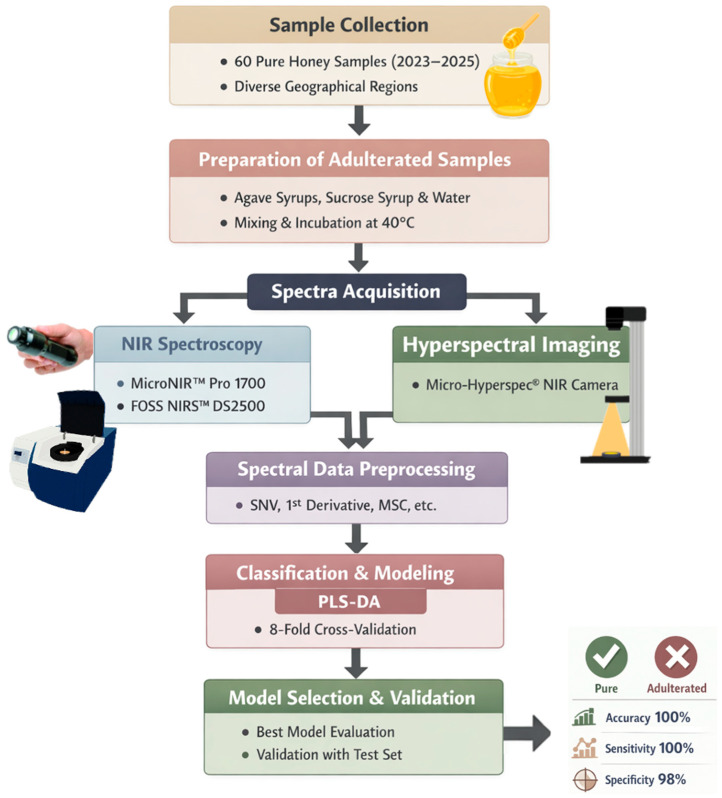
Schematic workflow for the portable and benchtop NIRS and hyperspectral imaging analysis.

The best model for each instrument was selected based on the aforementioned statistics and was then validated using the samples included in the validation set. Figure 3 represents a schematic overview of the experimental workflow used in the present study.

## 3. Results

### 3.1. Spectral Features

**Figure 4:**
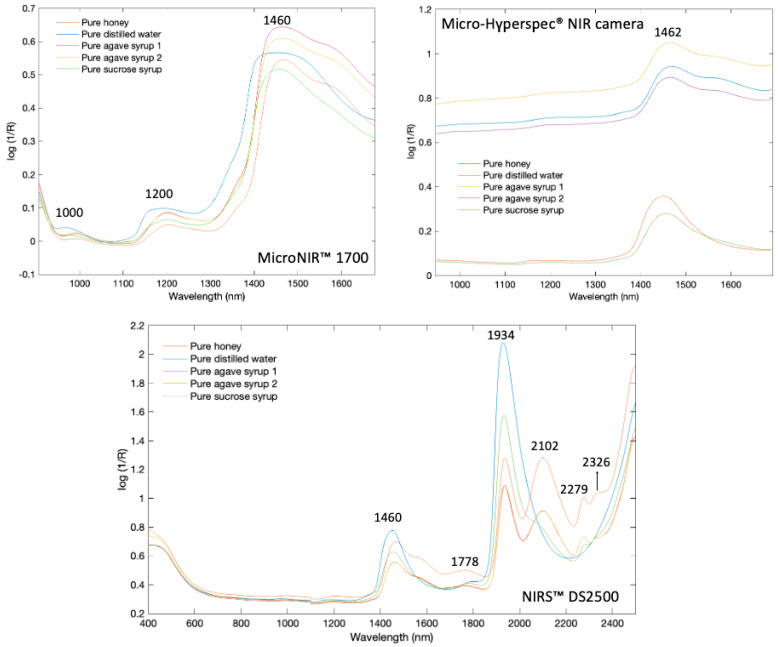
Raw spectra collection of all honey samples from MicroNIR™ 1700, Micro-Hyperspec^®^ NIR camera, and NIRS™ DS2500.

**Figure 5:**
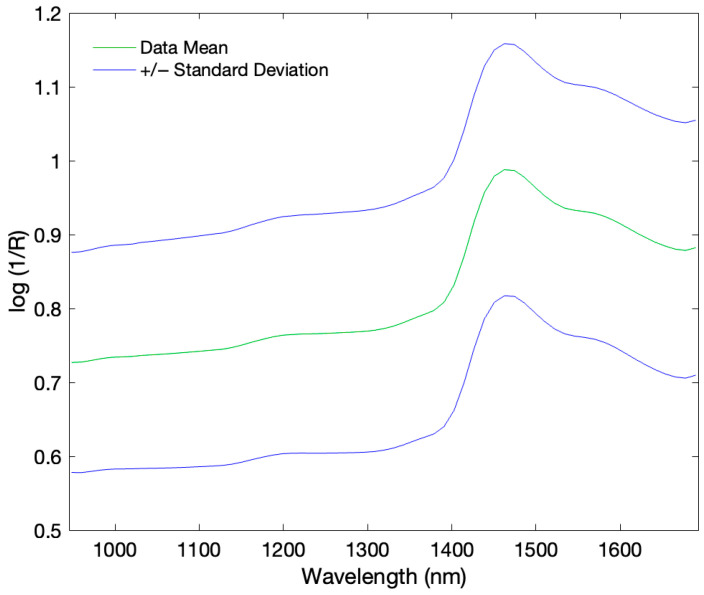
Mean spectrum of the analyzed samples with the corresponding standard deviation range, illustrating spectral variability of Micro-Hyperspec^®^ NIR camera.

The mean raw spectra of the pure honey samples and pure adulterants (sucrose syrup, distilled water, and dark and light agave syrups) obtained from MicroNIR™ 1700, NIRS™ DS2500, and Micro-Hyperspec^®^ NIR camera systems are shown in Figure 4. The mean spectrum, along with the corresponding standard deviation range obtained with the Micro-Hyperspec^®^ NIR camera, is provided in Figure 5.

Characteristic absorption bands (Log 1/R) were mainly observed in the NIR region. In the NIRS™ DS2500 spectra, key peaks appeared at 1460, 1778, 1928, 2102, 2279, and 2326 nm, corresponding to O-H stretching/bending, C-O stretching, and C-H/CH_2_ combinations, with water showing dominant features at 1467 and 1928 nm [10,38,39,40]. The most distinct separation between honey and adulterants occurred near 1925–1945 nm was attributed to O-H stretching and bending.

The NIRS™ DS2500 exhibited sharper peak features, particularly in the 1900–2500 nm region. This is consistent with its higher optical resolution (2 nm), which sets it apart from portable devices.

The MicroNIR™ 1700 detected consistent peaks between 1200 and 1467 nm (1467 nm for honey, 1460 nm for agave and sucrose syrups, and 1453 nm for water). The Micro-Hyperspec^®^ NIR camera revealed a single major feature at ~1462 nm, with observable spectral differences between dark and light agaves. Distilled water and sucrose syrup exhibited lower absorbance due to their high-water content and lack of organic compounds that enhance absorption in honey or agave syrup [41]. The lower number of detected peaks relative to other devices may be attributed to the hyperspectral camera’s limited spectral resolution. Overall, typical NIR absorptions in honey include ~1450 nm (O-H overtone), ~1765 nm (CH_2_ overtone), ~1940 nm (O-H stretch/bend), ~2100 nm (O-H deformation/C-O stretch), ~2280 nm (C-H stretch/deformation), and ~2345 nm (CH_2_ stretch/deformation) [42].

### 3.2. PCA Results

The Hotelling’s *T*^2^ limits were determined to be 8.08 for the MicroNIR™ 1700, 8.06 for the NIRS™ DS2500, and 6.13 for the Micro-Hyperspec^®^ NIR camera. The corresponding Q residual limits were calculated as 18.90 × 10^−4^ for the MicroNIR™ 1700, 3.13 × 10^−4^ for the NIRS™ DS2500, and 3.40 × 10^−4^ for the Micro-Hyperspec^®^ NIR camera. One pure honey sample exceeded the Hotelling’s *T*^2^ limit in both the MicroNIR™ 1700 and NIRS™ DS2500 datasets. This anomaly was likely caused by a reduced sample volume, resulting in low absorbance and the presence of bubbles, which compromised spectral quality. Consequently, this sample was removed from all datasets (MicroNIR™ 1700, NIRS™ DS2500, and Micro-Hyperspec^®^ NIR camera), reducing the total number of valid samples to 179. 

**Figure 6:**
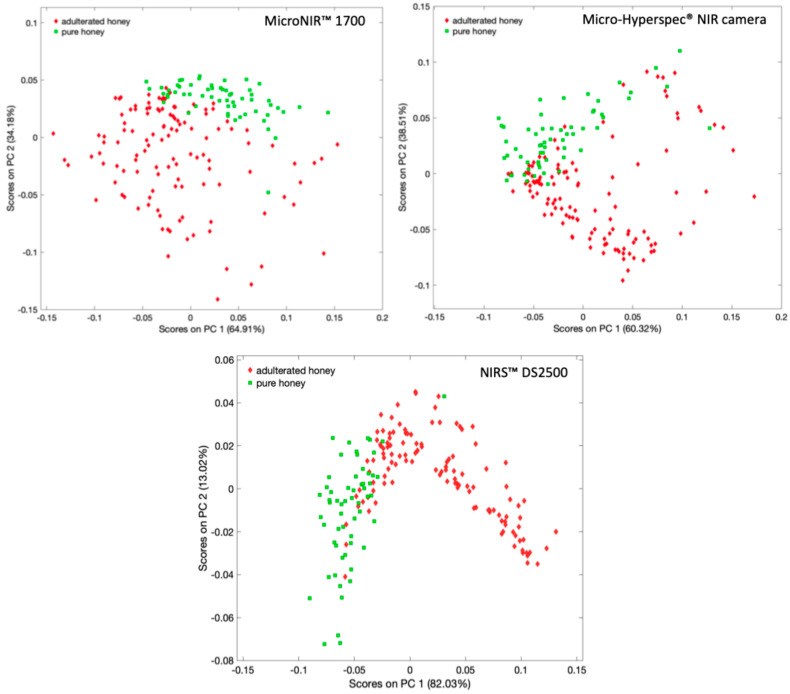
2D plot of first (*PC1*) versus second (*PC2*) principal component scores of the honey samples analyzed by MicroNIR™ 1700, Micro-Hyperspec^®^ NIR camera, and NIRS™ DS2500.

The first three components were selected for the MicroNIR™ 1700 dataset, while the first two components were selected for the Micro-Hyperspec^®^ NIR camera and the NIRS™ DS2500. The selection of *PCs* was justified by the cross-validation plot results, which showed an elbow in Root Mean Square Error of Calibration (*RMSEC*) and Root Mean Square Error of Cross-Validation (*RMSECV*) after the second and third components. This indicates that further *PCs* primarily represented noise rather than structural variance. Two-dimensional PCA score plots (*PC1* vs. *PC2*) obtained from MicroNIR™ 1700, NIRS™ DS2500, and Micro-Hyperspec^®^ NIR camera analyses demonstrate clear clustering of adulterated and pure honey samples (Figure 6). In all cases, *PC1* accounted for over 60% of the total variance in all instruments. The MicroNIR™ 1700 and NIRS™ DS2500 datasets exhibited more distinct separation between classes, indicating stronger discriminatory capability in the unsupervised space. In contrast, the Micro-Hyperspec^®^ NIR camera dataset showed slightly greater overlap between classes. Overall, the PCA results demonstrate that all three techniques are capable of capturing the variance associated with honey adulteration.

**Figure 7:**
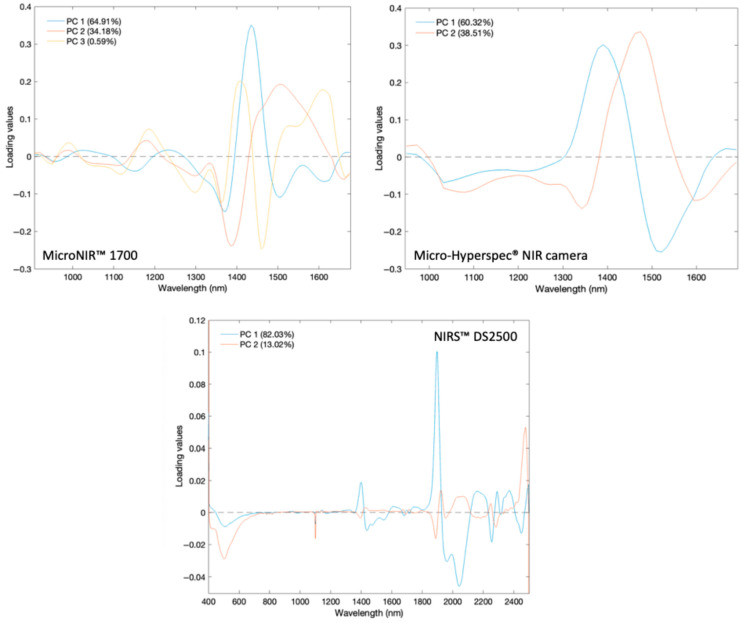
Loading values for the principal components (*PC1*, *PC2*, and *PC3*) of all honey samples analyzed by each device. The horizontal dashed line (*y* = 0) indicates the zero loading value.

Loading plots for the three instruments reveal that the most influential variables in class separation are located in spectral regions associated with water and carbohydrate absorptions (Figure 7). For the MicroNIR™ 1700 system, *PC1* (64.91%) shows a dominant peak at 1434 nm, *PC2* (34.18%) at 1508 nm, and *PC3* (0.59%) at 1403 nm; these are all within the O-H overtone and C-H combination regions [43]. The Micro-Hyperspec^®^ NIR camera displays similar trends, with *PC1* (60.32%) peaking at 1390 nm and *PC2* (38.51%) at 1474 nm, the latter is linked to higher overtone C-H/N-H absorptions [42]. The NIRS™ DS2500, which benefits from a broader spectral range, identifies multiple key bands: *PC1* (82.03%) at 1400, 1899, 2164, 2291, and 2372 nm and *PC2* (13.02%) at 1927 and 2473 nm. These encompass water O-H overtones, water combination bands, and C-H/C-O combination bands of sugars and other organic compounds [43,44]. These results suggest that moisture-related absorptions, alongside carbohydrate-associated bands, are responsible for most of the spectral variance between pure and adulterated honey samples.

### 3.3. Development of PLS-DA Models for the Discrimination of Pure and Adulterated Honey

**Figure 8:**
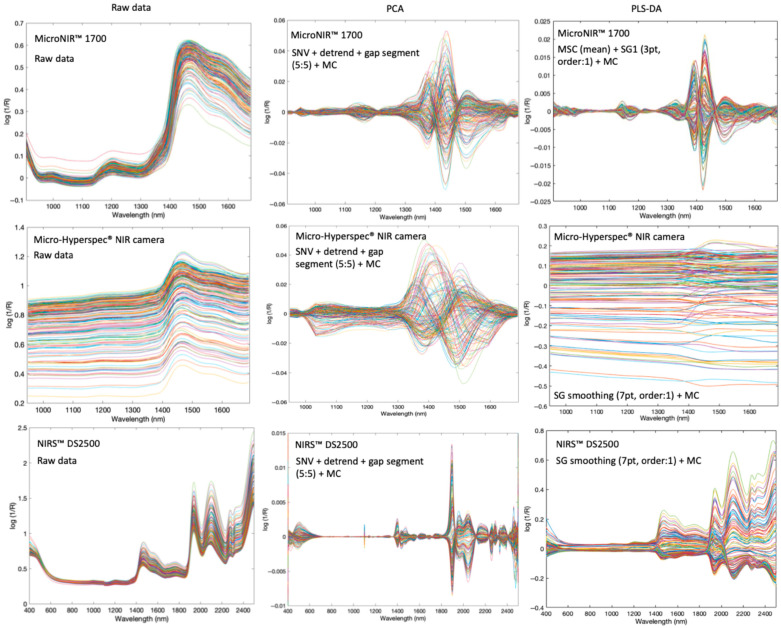
Raw data and pre-processing after PCA and PLS-DA for MicroNIR™ 1700, Micro-Hyperspec^®^ NIR camera, and NIRS™ DS2500.

The calibration set used to develop the classification models comprised 140 samples: 40 pure honey, 50 sucrose-adulterated honey, 25 agave-adulterated honey, and 25 water-adulterated honey. The optimal threshold values for classification were determined as 0.54, 0.68, and 0.61 for the MicroNIR™ 1700, the NIRS™ DS2500, and the Micro-Hyperspec^®^ NIR camera, respectively. The optimal pre-processing methods for each device were identified as MSC + SG first derivative (window size: 3 pt and polynomial order: 2) + MC for the MicroNIR™ Pro 1700, SG smoothing (7 pt and polynomial order: 1) + MC for the NIRS™ DS2500, and SG smoothing (7 pt and polynomial order: 1) + MC for the Micro-Hyperspec^®^ NIR camera (Table 4). All spectral profiles before and after preprocessing steps (SNV, DT, first derivative, and MC) are provided in Figure 8 to illustrate the reduction in scattering effects and baseline shifts.

Model optimization was guided by the minimum *RMSECV*, resulting in the selection of 3–4 latent variables (Table 4). Cross-validation metrics (Table 4), including sensitivity, specificity, and overall accuracy, indicate that all three devices performed robustly. In the context of food safety, adulterated honey was designated as the positive class, meaning that true positives corresponded to correctly identified adulterated samples. The results obtained with the three devices indicated a high capability to correctly identify pure honey samples, as reflected by their high accuracy. Sensitivity values, reflecting the correct detection of adulterated honey, were consistently high across the devices, particularly the MicroNIR™ Pro 1700 and the NIRS™ DS2500.

An in-depth study of the cross-validation results obtained from the developed models revealed device-dependent differences in misclassification rates. For the MicroNIR™ Pro 1700 device, one pure honey sample was misclassified as adulterated, while five adulterated samples were misclassified as pure. The misclassified adulterated samples comprised two 5% and one 20% sucrose-adulterated and two 10% light agave-adulterated samples. The classification model for the NIRS™ DS2500 achieved the best performance, with 100% accuracy, specificity, and sensitivity. All pure and adulterated samples were correctly identified without any misclassifications. For the Micro-Hyperspec^®^ NIR camera, eleven adulterated samples and ten pure honey samples were misclassified, corresponding to 89 correctly classified adulterated samples and 30 correctly classified pure samples. This indicates a higher misclassification rate compared with the other devices. 

**Figure 9:**
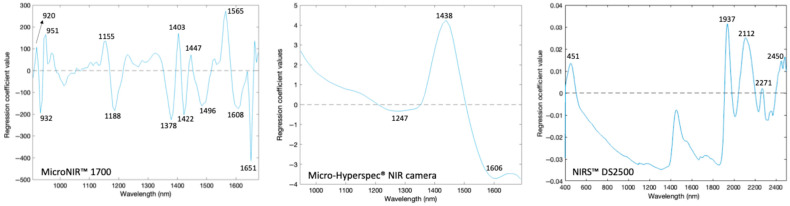
Regression coefficients of the best classification models developed to discriminate adulterated and pure honeys using MicroNIR™ Pro 1700, Micro-Hyperspec^®^ NIR camera, and NIRS™ DS2500.

The regression coefficient values of the best classification models revealed distinct spectral features that serve as key markers for distinguishing between pure and adulterated honey (Figure 9). For the MicroNIR™ Pro 1700, several prominent absorption peaks were observed at 930, 1155, 1403, 1447, and 1608 nm. These bands are associated with the O-H stretching overtones of water and sugars, as well as C-H stretching combinations of carbohydrates, both of which are highly sensitive to compositional changes induced by adulteration. Similarly, the Micro-Hyperspec^®^ NIR camera exhibited characteristic absorption features detected at 1247, 1438, and 1606 nm. The NIRS™ DS2500 identified peaks at 451, 1937, 2112, 2271, and 2450 nm, indicating broader spectral coverage and sensitivity, especially in regions affected by hydroxyl, carbohydrate, and minor nitrogen-containing compounds. These results are in strong agreement with literature reports. According to Frizon et al. [45], the spectral range of 1344–1699 nm is dominated by stretching and deformation vibrations of C-H, O-H, and N-H groups, which are abundant in phenolic acids and other bioactive compounds in honey. Furthermore, Cascant et al. [46] found that the bands in the 1360–1450 nm region were mainly associated with C-H and O-H vibrations. They also found that regions up to 1570 nm also include contributions from N-H overtones.

In addition to these assignments, the 451 nm waveband is associated with color-related compounds, reflecting variations in pigments and phenolic content in honey [47]. The spectral regions around 1900–1950 nm are also widely recognized as being associated with strong O-H combination bands, primarily related to water content and hydrogen bonding interactions in honey [47]. This region is critical for moisture evaluation and adulteration detection. Furthermore, the regions between 2000 and 2400 nm are attributed to combination vibrations of C-H, O-H, and C-O bonds, which are strongly linked to the carbohydrate structure, including glucose, fructose, and sucrose [42]. These bands are particularly important for distinguishing between authentic and adulterated honey, as they reflect variations in sugar composition and the way in which sugars interact with other molecules within the honey matrix.

**Figure 10:**
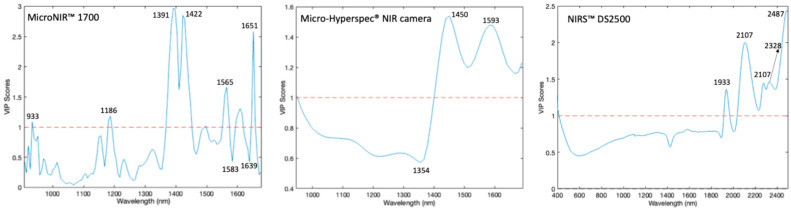
VIP scores of the best classification models developed to discriminate adulterated and pure honeys using MicroNIR™ Pro 1700, Micro-Hyperspec^®^ NIR camera, and NIRS™ DS2500. The blue line represents the VIP score values, and the red dashed line indicates the threshold of 1.0.

Variable Importance in Projection (VIP) scores determine which bands have the most influence on the models. Values close to or greater than one indicate significant contributions. For the MicroNIR™ Pro 1700 system, the VIP scores confirmed that the model relied on the 1390–1450 nm and 1600–1650 nm regions. These correspond to the first overtones of the O-H stretching (water/carbohydrates) and C-H vibrations. This ensures that the classification is based on chemical signatures rather than noise (Figure 10). Similarly, the Micro-Hyperspec^®^ NIR system identified key contributions at 1450 nm and ~1600 nm, which correspond to O-H and C-H-related absorptions. By contrast, the NIRS™ DS2500 system, which covers a broader spectral range, revealed additional important regions not only around 1900 nm (linked to water absorption) but also in the 2000–2500 nm region. This region is strongly associated with carbohydrate structures such as glucose and fructose.

The overlap between high VIP values and prominent regression coefficients demonstrates that the models successfully identified chemically significant spectral features related to moisture and sugar content, which supports their reliability in assessing honey quality and detecting adulteration. Findings from the MicroNIR™, the NIRS™ DS2500, and the Micro-Hyperspec^®^ NIR camera consistently highlight the 1400–1600 nm region, reflecting O-H and C-H bond interactions, as a critical diagnostic window for honey authentication. Furthermore, the NIRS™ DS2500 exhibits valuable sensitivity above 2300 nm, capturing C-H stretch and deformation combination bands, thereby enhancing adulteration detection.

Therefore, combining instrumental results with established vibrational assignments reveals that NIR spectroscopy, in handheld, imaging, or benchtop forms, provides robust and complementary insights for the classification and adulteration detection of honey.

### 3.4. External Validation of the PLS-DA Models Devised

To assess the robustness and generalizability of the models, the external validation set comprised 29 independent samples. This set included nine pure honeys, ten sucrose-adulterated honeys, five agave-adulterated honeys, and five water-adulterated honeys.

**Figure 11:**
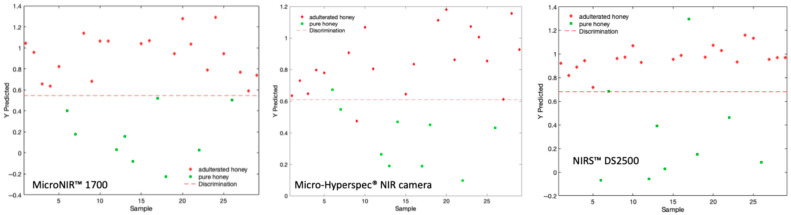
External validation of best models of pure vs adulterated honeys using MicroNIR™ 1700, NIRS™ DS2500, and Micro-Hyperspec^®^ NIR camera.

The results of the external validation are shown in Table 5. For all three devices, adulterated honey samples were consistently predicted above the classification threshold, while pure honey samples were predicted below, indicating successful discrimination across different instruments. The MicroNIR™ Pro 1700 showed the clearest separation: adulterated samples were strongly above the threshold, and pure honey samples were well below it. This resulted in 100% accuracy, specificity, and sensitivity, highlighting the superior performance and robustness of the device (Figure 11).

The Micro-Hyperspec^®^ NIR camera demonstrated effective discrimination, although with smaller classification margins for pure honey, resulting in two samples being misclassified. These were 10% light agave syrup adulteration and one pure honey sample. These results indicate that agave syrup is particularly challenging to detect at low levels due to its sugar composition, which closely resembles authentic honey. According to Ciursa and Oroian [48], this difficulty arises from the high fructose content of agave syrup and its elevated fructose/glucose ratio. The external validation of Micro-Hyperspec^®^ NIR camera yielded 93.10% accuracy, 95% sensitivity, and 88.00% specificity, reflecting robust overall performance with only a slightly higher susceptibility to borderline misclassifications.

The NIRS™ DS2500 also demonstrated high predictive performance, matching the Micro-Hyperspec^®^ NIR camera with an accuracy of 93.10%. It achieved a sensitivity of 100%, correctly identifying all adulterated samples; however, its specificity was 78.00% due to two pure honey samples being misclassified as adulterated. While the NIRS™ DS2500 is exceptionally reliable at ensuring the detection of adulterated honey, it may be more sensitive to natural variations in pure honey profiles. This sensitivity could lead to tighter or more overlapping classification margins compared to the MicroNIR™ Pro 1700.

## 4. Discussion

The results demonstrate that the MicroNIR™ Pro 1700 provided the most reliable performance (sensitivity, specificity, and accuracy) in distinguishing pure from adulterated honey, achieving the highest sensitivity, specificity, and accuracy (100%). Notably, in cross-validation, the NIRS™ DS2500 delivered the best classification results, while the MicroNIR™ Pro 1700 provided the robust prediction model, reaching 100% sensitivity, specificity, and accuracy. In addition, the Micro-Hyperspec^®^ NIR camera exhibited slightly higher misclassification rates, particularly for adulterated samples. This difference, corresponding to an approximate 10% reduction in classification accuracy, can be primarily attributed to the non-contact nature of the HSI camera, which introduces additional variability due to atmospheric interference, ambient noise, and path-length fluctuations. 

Despite these limitations, the performance of the HSI system remains competitive. The application of spatial averaging within the ROI significantly enhances the signal-to-noise ratio and enables the capture of sample heterogeneity. As a result, the system effectively detects key spectral features associated with O-H and C-H overtones and achieves a high classification accuracy of 90%. These findings are consistent with previous studies reporting NIR-based honey adulteration detection accuracies ranging from 92.5% to 95% [25,26], depending on the adulterant type, spectral range, and modeling approach. The observed differences highlight the trade-off between spectral precision and spatial information, where point-based spectrometers offer higher spectral stability, while HSI provides additional spatial insights.

The external validation results further confirm the robustness of the developed models. The MicroNIR™ Pro 1700 demonstrated consistent performance across independent measurements, reinforcing its suitability as a rapid and reliable tool for routine honey authentication. In contrast, while the Micro-Hyperspec^®^ NIR system and the NIRS™ DS2500 remained highly effective, they showed a slightly higher susceptibility to borderline misclassifications, particularly in the case of agave syrup adulteration. This may be explained by the spectral similarity between agave syrup and natural honey, which reduces class separability in the overlapping O-H absorption regions.

Although the results are promising, several limitations should be considered. The adulteration scenarios investigated in this study were limited to sucrose syrup, agave syrup, and distilled water at controlled concentrations. While these adulterants are relevant and commonly reported, real-world honey fraud often involves a wider range of syrups (e.g., corn and rice syrups) and more complex multi-component adulteration strategies. For instance, adulterants with similar chemical compositions could generate overlapping spectral features, potentially reducing classification accuracy by an estimated 5–15%, particularly near decision boundaries. Furthermore, multi-component adulteration may lead to increased model uncertainty and a higher likelihood of false negatives. Therefore, while the developed models demonstrate strong performance under controlled conditions, their generalizability to more complex and diverse adulteration scenarios requires further investigation.

From a practical perspective, the proposed spectroscopic techniques offer significant advantages over conventional analytical methods. Traditional approaches, such as high-performance liquid chromatography and isotope ratio mass spectrometry, typically involve extensive sample preparation, require skilled personnel, and are time-consuming. In contrast, NIR-based techniques enable rapid, non-destructive analysis with minimal or no sample preparation, delivering results within seconds. This substantially reduces analysis time and operational costs while increasing throughput, making these methods highly suitable for large-scale screening applications.

In particular, portable devices such as the MicroNIR™ Pro 1700 provide a practical solution for on-site analysis in supply chains, production facilities, and regulatory inspection points. Their ease of use and rapid response capability make them ideal for routine quality control and fraud prevention. On the other hand, imaging systems offer a complementary advantage by enabling spatially resolved analysis. This capability is particularly valuable for detecting heterogeneous adulteration patterns or localized variations within bulk samples, which cannot be captured using point-based measurements.

Future research should focus on expanding the dataset to include a wider variety of adulterants and their combinations at different concentration levels. Increasing the diversity of the spectral library, along with the integration of advanced machine learning techniques, could further improve model robustness and generalizability. Ultimately, these developments could support the development of universal, field-deployable screening systems for comprehensive honey authenticity assessment, contributing to enhanced food safety, regulatory enforcement, and consumer confidence.

## 5. Conclusions

This study demonstrates that NIR spectroscopy and hyperspectral imaging systems are effective and reliable tools for detecting honey adulteration. External validation showed 100% accuracy for the portable MicroNIR™ Pro 1700 and 93.10% accuracy for the Micro-Hyperspec^®^ NIR camera and for the benchtop NIRS™ DS2500, confirming the robustness of developed models. Beyond analytical success, the ability to rapidly and non-destructively detect adulteration is crucial for preventing food fraud, ensuring fair economic practices, and protecting consumer trust and health. Among the evaluated systems, the MicroNIR™ Pro 1700 stands out as the most practical for industrial applications due to its portability and speed. Meanwhile, the Micro-Hyperspec^®^ NIR camera provides added value for research, and the NIRS™ DS2500 remains suitable for laboratory-based quality control. Overall, these technologies have strong potential to support the assurance of authenticity and sustainable development in the honey industry. Future studies may extend the developed models to more complex and unknown adulteration scenarios to enhance their applicability in real market conditions further.

## Figures and Tables

**Table 1:** Characterization of the honey sample set.

Region	Country	Type	Year	Number of Samples
Tbilisi	Georgia	Flower	2024	1
Córdoba	Spain	Flower	2024	4
		Flower (eucalyptus)	2024	2
		Flower (rosemary)	2024	1
Ağrı	Türkiye	Flower	2024	2
Ankara		Flower	2024	1
Bayburt		Flower	2025	1
Erzincan		Flower	2024–2025	3
Erzurum		Flower	2024–2025	4
Eskişehir		Flower	2025	1
Giresun		Flower	2025	1
Gümüşhane		Flower	2025	1
Hakkari		Flower	2025	1
İstanbul		Flower	2024–2025	16
		Pine	2025	1
Kastamonu		Chestnut	2025	1
Kayseri		Flower	2025	1
Muğla		Flower	2025	1
		Pine	2025	1
Ordu		Flower	2025	2
		Mad	2025	1
Rize		Flower	2023–2024	2
Sakarya		Chestnut	2025	1
Siirt		Flower	2025	2
Sivas		Flower	2025	2
Van		Flower	2025	1
Yalova		Chestnut	2025	1
		Flower	2023–2024	2
Yozgat		Flower	2025	2

**Table 2:** Number of pure and adulterated honey samples used in the study.

Adulterant Type	Concentration Levels (*w*/*w*%)	Number of Adulterated Honey Samples Per Concentration Level	Base Pure Honey Samples (IDs)	Total Samples
Pure Honey	100	0	P01-60	60
Agave Syrup 1 (dark)	5, 20, 40	5	P01-05	15
Agave Syrup 2 (light)	10, 30, 50	5	P06-10	15
Sucrose Syrup	5, 10, 20, 30, 40, 50	10	P01-10	60
Distilled Water	5, 10, 20	10	P01-10	30
Total	–	–	–	180

P01–P10 refer to the specific 10 honey samples selected for the adulteration process, representing diverse geographical origins (Black Sea, Marmara, and Central Anatolia regions of Türkiye; and Cordoba of Spain) and floral sources (Monofloral: Rosemary, Eucalyptus, Orange blossom; Multifloral: Wildflower).

**Table 3:** Instrumental properties of portable and benchtop NIR spectroscopy and HSI camera.

Devices	MicroNIR™ 1700	NIRS™ DS2500	Micro-Hyperspec^®^ NIR Camera
Manufacturer and model	VIAVI/Pro 1700	FOSS/DS2500	HEADWALL
Instrument type	Portable/Handheld	Benchtop	On-line
Detector	InGaAs	Silicon and PbS	InGaAs
Spectral range (nm)	908–1676	400–2500	889–1702
Acquisition Time	Low	Medium	High
Portability/Ease of use	High; handheld, easy on-site measurement	Low; with mobile parts, requires a lab setting	Low; benchtop, complex alignment and imaging
Cost Level	Low	Medium	High
Industrial suitability	Ideal for on-line/at-line quality control in factories; offering rapid, robust and non-destructive detection.	Suitable for lab-based quality control; offering high precision with moderate throughput in controlled environments.	Best for research or detailed mapping; but less practical for high-throughput industrial quality control

**Table 4:** Model classification performance for cross-validation on different devices for detecting adulterated honey.

Devices	MicroNIR™ Pro 1700	Micro-Hyperspec^®^ NIR Camera	NIRS™ DS2500
Pre-processing	MSC + SG1 (ws: 3 pt; order: 2) + MC	SG smoothing (7 pt; order: 1) + MC	SG smoothing (7 pt; order: 1) + MC
LV	4	3	4
Accuracy	95.71%	85.00%	100%
Specificity	97.00%	75.00%	100%
Sensitivity	95.00%	89.00%	100%

LV: latent variables, MC: mean center, MSC: multiplicative scattering correction, pt: point, SG: Savitzky-Golay, SG1: SG first derivative, ws: window size.

**Table 5:** Model classification performance for prediction on different devices for detecting adulterated honey.

Devices	MicroNIR™ Pro 1700	Micro-Hyperspec^®^ NIR Camera	NIRS™ DS2500
Accuracy	100%	93.10%	93.10%
Specificity	100%	88.00%	78.00%
Sensitivity	100%	95.00%	100%

## Data Availability

The data presented in this study are available on request from the corresponding authors.
